# ELABELA promotes the migration and homing of bone marrow mesenchymal stem cells to myocardial injury sites through the ERK1/2/miR-299a-5p/Exo70 pathway

**DOI:** 10.3389/fphar.2025.1541869

**Published:** 2025-02-03

**Authors:** Jing-Yu Hou, Hao Wu, Shuang-Mei Li, Xiao-Jing Li, Shu-Jun Yang, Xu-Xiang Chen, Chang-Qing Zhou, Hui-Bao Long, Hai-Dong Wu, Jia-Ying Fu, Ya-Jie Guo, Tong Wang

**Affiliations:** ^1^ Department of Emergency, The Eighth Affiliated Hospital of Sun Yat-sen University, Shenzhen, Guangdong, China; ^2^ Department of Emergency, Sun Yat-sen Memorial Hospital of Sun Yat-sen University, Guangzhou, Guangdong, China

**Keywords:** Elabela, bone marrow mesenchymal stem cells, migration and homing, myocardial injury, Exo70

## Abstract

**Background:**

Bone marrow mesenchymal stem cells (BMSCs) hold promise for repairing myocardial injury following acute myocardial infarction (AMI), but their clinical application is hindered by poor migration, homing efficiency, and survival rates. Previously, we demonstrated that ELABELA (ELA), a small peptide, enhances the survival of rat BMSCs under hypoxia-reoxygenation (H/R) conditions by activating ERK1/2. However, the role of ELA in promoting BMSCs migration and homing to injured cardiomyocytes remains unclear.

**Methods:**

Primary BMSCs and neonatal rat ventricular myocytes (NRVMs) were isolated and cultured. NRVMs were exposed to H/R to mimic the microenvironment of AMI in vitro. The migration of BMSCs toward the injured myocardium was assessed in different treatment groups using transwell and chemotaxis assays. Additionally, in vivo studies were performed using a rat myocardial infarction/reperfusion injury (MI/RI) model with DIR-labeled BMSCs. Cardiac repair was evaluated through fluorescence imaging, echocardiography, and histological analysis. Transcriptome sequencing and bioinformatics analysis were employed to identify and validate the mechanisms by which ELA promoted the migration of BMSCs. A dual luciferase assay was used to investigate the interaction between Exo70 and miR-299a-5p. Subsequently, a series of experimental procedures were performed, including sequential silencing of APJ or Exo70, overexpression of miR-299a-5p, inhibition of ERK1/2 phosphorylation, assessment of BMSCs migration through transwell and scratch assays, detection of F-actin polymerization via immunofluorescence, and evaluation of the expression levels of each factor using qPCR and Western blotting.

**Results:**

In vitro, the migration ability of ELA-pretreated BMSCs was significantly augmented in the H/R environment. ELA pretreatment effectively heightened the homing capacity of BMSCs to the site of myocardial injury and their proficiency in repairing myocardial damage in vivo. Transcriptome sequencing revealed upregulation of Exo70 in ELA pretreated BMSCs, which promoted F-actin polymerization and migration. Overexpression of miR-299a-5p reduced Exo70 expression and impaired BMSCs migration. ELA also activated ERK1/2 phosphorylation, while inhibition of ERK1/2·with U0126 abrogated F-actin polymerization and migration, increasing miR-299a-5p levels and reducing Exo70.

**Conclusion:**

ELA enhances BMSCs migration and homing to injured cardiomyocytes by activating the APJ receptor, promoting ERK1/2 phosphorylation, downregulating miR-299a-5p, and upregulating Exo70, providing a potential therapeutic strategy for improving stem cell-based cardiac repair.

## 1 Introduction

Cardiovascular diseases (CVDs) are a leading cause of morbidity and mortality worldwide, with acute myocardial infarction (AMI) being one of the most prevalent and clinically challenging forms. Reperfusion, a commonly employed clinical intervention for AMI, has the potential to trigger a series of adverse reactions and worsen myocardial damage, ultimately resulting in increased mortality rates (Barrere-Lemaire et al., 2024; Heusch, 2020). Thus, it is critical to find strategies that not only mitigate reperfusion injury but also enhance myocardial repair. While conventional pharmaceutical agents and surgical procedures can partially or fully reinstate blood circulation and mitigate inflammation, they are unable to salvage the functional viability of myocardial cells within the infarcted region (Ji et al., 2017). With the development of cell therapy technology, stem cells have received much attention from researchers because of their ability to self-renew, multidirectional differentiation, and paracrine signaling under appropriate conditions (Zhang S. et al., 2021). Among them, bone marrow mesenchymal stem cells (BMSCs) are the most widely studied and relatively easy to obtain mesenchymal stem cells (MSCs). The transplantation of BMSCs after AMI has been shown to be effective in limiting infarct areas and repairing myocardial damage in animal and clinical trials (Attar et al., 2021; Jiang et al., 2021; Cui et al., 2024). However, clinical application remains limited, as only a tiny number of BMSCs migrate to the local area of the infarcted myocardium and participate in limited post-MI tissue repair when BMSCs are infused locally in the non-infarcted myocardium (e.g., during intravenous infusion or intramyocardial injection) (Golpanian et al., 2015; Saeedi et al., 2021). Enhancing the homing capacity of BMSCs could improve their therapeutic potential by increasing their migration to the damaged site and promoting the secretion of pro-tissue regenerative chemokines, cytokines, and growth factors (Bui et al., 2021).

Cell migration and homing are crucial for the therapeutic efficacy of BMSCs, and the prime mover affecting directed cell migration requires the coordination of actin assembly and membrane remodeling (Andersen et al., 2023; Faix and Rottner, 2022). Exocyst complex component 7 (Exoc7, Exo70) is an essential component of the extracellular secretory complex, which interacts directly with the Actin-related proteins 2/3 (Arp2/3) complex. This core nucleating factor generates the branching actin network and promotes F-actin polymerization for cell morphogenesis and migration (Sveeggen et al., 2023; Ntantie et al., 2017). Identifying ways to enhance F-actin polymerization could significantly improve BMSCs migration.

ELABELA (ELA, Toddle) is an endogenous ligand for Angiotensin receptor-like 1 (APLNR, APJ) in early embryonic development, which has a similar sequence to apelin. Still, its receptor affinity is about five times that of apelin. ELA has been found to be promising and relatively safe for treating cardiovascular disease (Liu et al., 2020; Zhou and Wu, 2022; Chapman et al., 2023). Our previous studies have found that BMSCs pretreated with ELA could be protected by reducing apoptosis and promoting proliferation in a harsh environment of ischemia and hypoxia (Chen et al., 2022; Fu et al., 2020). However, the molecular mechanisms by which ELA regulates the migration of BMSCs remain unclear. In this study, we aimed to investigate whether ELA can promote the migration and homing of BMSCs to repair injured myocardial tissue by affecting F-actin polymerization.

Through both *in vivo* rat models and *in vitro* cell experiments, we aim to investigate whether ELA pretreatment enhances the migration and homing capacity of BMSCs to the damaged myocardial site, and promotes tissue repair in reperfused myocardial injury. Specifically, we hypothesize that this effect is mediated through the downregulation of microRNA-299a-5p (miR-299a-5p) via ERK1/2 phosphorylation, which leads to increased Exo70 expression and subsequent F-actin polymerization. The goal of this study is to elucidate the molecular mechanisms underlying BMSCs migration, which may improve BMSCs transplantation therapy for AMI. Additionally, the findings could contribute to the development of ELA-based therapeutic strategies, facilitating the clinical application of BMSCs transplantation in AMI treatment.

## 2 Methods

### 2.1 Ethics statement

Male Sprague-Dawley (SD) rats (80∼120 g and 200∼250 g, SPF grade) and newborn SD suckling rats (aged 1–3 days, SPF grade) were purchased from the Zhuhai BesTest Bio-Tech Co., Ltd. (Zhuhai, China), with license number SYXK (Guangdong) 2020-0051. All animal experiments were approved by the Animal Ethics Committee of the Eighth Affiliated Hospital of Sun Yat-sen University (Approval number: 2022-081-01).

### 2.2 Chemicals

ELA, a synthetic polypeptide consisting of 32 amino acids (sequence: QRPVNLTMRRKLRKHNCLQRRCMPLHSRVPFP), was purchased from GL Biochem Co. Ltd. (Shanghai, China). The ELA powder had a purity of 95.31% and was stored at −20°C. Before use, ELA was dissolved in phosphate-buffered saline (PBS) at a final concentration of 5 μM and filtered through a 0.22 μm filter.

### 2.3 Cell isolation and culture

BMSCs were isolated from the femurs and tibias of SD rats (80∼120 g) as previously described (Chen et al., 2022). BMSCs were cultured in a complete culture medium consisting of low-glucose Dulbecco’s modified Eagle’s medium (GIBCO, United States), supplemented with 10% fetal bovine serum (GIBCO, United States) and 1% penicillin/streptomycin (HyClone, United States). After 24 h, non-adherent cells were discarded, and the medium was replaced. When cells reached 80%–90% confluence, they were passaged at a 1:2 ratio. All experiments were conducted using BMSCs at passage 3 (P3). As previously reported by our research group (Fu et al., 2020), phenotyping of the BMSCs was confirmed via fluorescence-activated cell sorting revealing that the cells were positive for CD44 and CD29 but negative for CD34.

Neonatal rat ventricular myocytes (NRVMs) were isolated from newborn SD suckling rats (aged 1–3 days) following the protocol outlined in our earlier reports (Chen et al., 2014). The collected hearts were cut into 1–2 mm^3^ and digested with 0.25% trypsin solution (without EDTA) (Beyotime, China) at 37°C for 10 min. The supernatant fractions were discarded, and the remaining tissue samples were further digested using 0.1% type II collagenase (Sigma-Aldrich, United States) for 5-6 times (10 min each) in a 37°C water bath. The cell suspension was collected and filtered through a 70 μm cell strainer and centrifuged at 1,500 rpm for 5 min. The cell pellet was resuspended in high-glucose Dulbecco’s Modified Eagle Medium (DMEM; GIBCO, United States) containing 10% fetal bovine serum (GIBCO, United States) and 1% penicillin-streptomycin. Most fibroblasts were removed from the suspension by two successive adherence steps on plastic (approximately 45 min each). Any remaining fibroblasts in the culture medium containing NRVMs were inhibited using Bromodeoxyuridine (BrdU, 0.1 mM final concentration; Sigma-Aldrich, United States). NRVMs were cultured in a 6-well plate at a density of 5 × 10^5^ cells/well, with media changes every 2–3 days for subsequent experiments after 5–6 days.

### 2.4 Animal grouping and surgery

SD rats (200–250 g) were ear-tagged by trained technicians using a metal applicator under sterile conditions and local anesthesia. Efforts were made to minimize stress and prevent injury. After ear tagging, rats were acclimated individually in SPF rooms for a week, with controlled temperature (24°C ± 2°C), humidity (50% ± 10%), and a 12-hour light-dark cycle, with *ad libitum* access to food and water. Daily health checks were conducted, and any rats showing signs of ill health were removed from the study. After acclimation, all rats were healthy, showing no abnormal behavior or ear infections.

As in previous studies (Jiang et al., 2023; Liu et al., 2019), the rats were anesthetized and subjected to localized myocardial ischemia by ligating the left anterior descending branch of the coronary artery. After 45 min, the ligature was released, and reperfusion was initiated for 1 hour. Electrocardiography (ECG) (RM6240E, Chengdu, China) was employed to assess the ischemic cardiac tissue and confirm the successful establishment of the myocardial infarction/reperfusion injury (MI/RI) model. A total of 32 male SD rats were randomly divided into four groups (n = 8 per group): (1) sham group: rats underwent thoracotomy without coronary artery ligation; (2) MI/RI group: rats underwent left anterior descending branch ligation, followed by 1 hour of reperfusion and were injected with 500 μL of PBS via the tail vein; (3) MI/RI + BMSCs group: rats received 500 μL of PBS containing BMSCs (4 × 10^6^ cells/mL) via intravenous injection following MI/RI; (4) MI/RI + ELA + BMSCs group: rats received 500 μL of PBS containing BMSCs pre-treated with ELA (4 × 10^6^ cells/mL) via intravenous injection following MI/RI. In all *in vivo* experiments, the BMSCs, whether pre-treated with ELA or not, were stained with DiR overnight.

### 2.5 *In vivo* imaging of DIR fluorescent dyes

DiR (DiIC18(7), Invitrogen, United States) was a lipophilic dye used for cell tracking in live imaging. Following the manufacturer’s instructions, *in vitro* staining was performed using a final concentration of 10 μM. SD rats (200–250 g) were anesthetized and injected with DiR-stained BMSCs or DiR-stained ELA pre-treated BMSCs through the tail vein, in accordance with their respective groups. Live imaging was immediately conducted using a small animal living imaging system (Tanon ABL X6pro, Shanghai, China) with an excitation filter of 750 nm and an emission filter of 780 nm. The Tanon imaging analysis system (Tanon, Shanghai, China) was utilized to acquire imaging images at 0 h, 3 h, and 24 h, and the total radiant efficiency of the heart was assessed and compared. Upon the rats’ euthanasia after 28 days, the hearts were extracted and the average radiant efficiency was measured using an IVIS Spectrum imaging system (PerkinElmer, United States).

### 2.6 Echocardiography

Cardiac function and therapeutic efficacy of BMSCs transplantation in myocardial injury were assessed at 14 and 28 days post-modeling using a real-time molecular imaging system (Vevo2100, Visualsonics, Canada). M-mode ultrasound was employed, with cardiac function measurements taken by ultrasound physicians in a double-blind manner. The parameters assessed included left ventricular internal diameter in systole (LVIDs), left ventricular internal diameter in diastole (LVIDd), Left ventricular ejection fraction (LVEF), left ventricular fractional shortening (LVFS), left ventricular end-systolic volume (LVESV), and left ventricular end-diastolic volume (LVEDV). These measurements were calculated using the Teichholtz formula over three consecutive cardiac cycles. The echocardiographic images were analyzed using Vevo 2100 software (Visualsonics, Canada).

### 2.7 HE and masson staining

Fresh heart tissue from SD rats was processed by fixing, dehydrating, embedding in paraffin, sectioning, and subsequently subjected to HE and Masson staining. HE staining involved the removal of paraffin and rehydration of the tissue sections using xylene and alcohol, followed by hematoxylin and eosin staining to highlight the cell nuclei and cytoplasm, respectively. The stained sections were then dehydrated through graded alcohol solutions and mounted with neutral gum. The slides were examined using an Aperio GT 450 scanner (Leica Biosystems, United States) and analyzed with ImageScope software (Aperio ImageScope, v12.4.6.5003). Masson staining procedure entailed the removal of paraffin and rehydration of heart tissue sections that had been embedded in paraffin. The sections were stained using the Masson tricolor staining kit (Servicebio, Wuhan, China). The stained sections were dehydrated, mounted, examined under a microscope (Nikon, Japan), and scanned (3DHISTECH, Hungary). The quantification of collagen protein content in the samples was conducted using ImageJ software (ImageJ 1.53t, United States).

### 2.8 Small interfering RNA (siRNA) transfections

BMSCs were cultured in medium without penicillin/streptomycin. The siRNA targeting APJ (siRNA-APJ), siRNA-APJ negative control (NC), siRNA-Exo70, siRNA-Exo70 NC, miR-299a-5p mimic, mimic NC, miR-299a-5p inhibitor, and inhibitor NC were purchased from RiboBio (RiboBio Co., Ltd., Guangzhou, China). The sequences were listed in Table 1. According to the manufacturer’s instructions, BMSCs were transfected using Lipofectamine RNAi Max Reagent (ThermoFisher, United States).

**TABLE 1:** Sequences of cell transfection.

Name	Sequences (5′-3′)
siRNA-APJ	GCC​TCA​GCT​TTG​ACC​GAT​A
siRNA-Exo70-1	CTA​CCA​AGA​AGC​CCA​TCA​A
siRNA-Exo70-2	CCA​GAC​AAG​GAG​TAC​AAC​A
siRNA-Exo70-3	CGG​AGA​AGA​TCA​TCA​GAG​A
miR-299a-5p mimic	F: UGGUUUACCGUCCCACAUACAU R: ACCAAAUGGCAGGGUGUAUGUA
mimic NC	F: UUUGUACUACACAAAAGUACUG R: AAACAUGAUGUGUUUUCAUGAC
miR-299a-5p inhibitor	AUG​UAU​GUG​GGA​CGG​UAA​ACC​A
inhibitor NC	CAG​UAC​UUU​UGU​GUA​GUA​CAA​A

### 2.9 Hypoxia reoxygenation-neonatal rat ventricular myocytes (H/R- NRVMs) model and BMSCs treatments

NRVMs were rinsed twice with PBS and incubated in low-glucose DMEM with serum-free medium (0% serum) in a hypoxia incubator chamber (STEMCELL, Canada) containing 1% O_2_, 94% N_2_, and 5% CO_2_ for 12 h to induce hypoxic injury. Cells were then treated with 10% serum and reoxygenated for 12 h in a 5% CO_2_ incubator at 37°C. The H/R-NRVMs medium was harvested and centrifuged at 3,000 rpm for 5 min to collect the supernatant, while NRVMs were cultured in low-glucose complete medium for 12 h.

A total of 22 groups were set up with different treatments: (1) siAPJ group: BMSCs transfected with siRNA-APJ; (2) siAPJ NC group: BMSCs transfected with siRNA-APJ NC; (2) siExo70 group: BMSCs transfected with siRNA- Exo70; (4) siExo70 NC group: BMSCs transfected with siRNA- Exo70 NC; (5) mimic NC: BMSCs transfected with NC mimic; (6) miR-299a-5p mimic: BMSCs transfected with miR-299a-5p mimic; (7) inhibitor NC: BMSCs transfected with inhibitor NC; (8) miR-299a-5p inhibitor: BMSCs transfected with miR-299a-5p inhibitor; (9) blank group: BMSCs without any treatment (negative control); (10) ELA group: BMSCs treated with 5 μM ELA for 24 h; (11) control group: BMSCs cultured in NRVMs medium for 12 h; (12) H/R group: BMSCs cultured in H/R-NRVMs medium for 12 h; (13)H/R + ELA group: BMSCs preconditioned with 5 μM ELA and cultured in H/R-NRVM medium for 12 h; (14) H/R + ELA + siAPJ group: BMSCs transfected with siRNA-APJ before treatment with 5 μM ELA; (15) H/R + ELA + siAPJ NC group: BMSCs transfected with siRNA-APJ NC before treatment with 5 μM ELA; (16) H/R + ELA + siExo70 group: BMSCs transfected with siRNA- Exo70 before treatment with 5 μM ELA; (17) H/R + ELA + siExo70 NC group: BMSCs transfected with siRNA-Exo70 NC before treatment with 5 μM ELA; (18) H/R + ELA + mimic NC: BMSCs transfected with NC mimic before treatment with 5 μM ELA; (19) H/R + ELA + miR-299a-5p mimic: BMSCs transfected with miR-299a-5p mimic before treatment with 5 μM ELA; (20) H/R + ELA + inhibitor NC: BMSCs transfected with inhibitor NC before treatment with 5 μM ELA; (21) H/R + ELA + miR-299a-5p inhibitor: BMSCs transfected with miR-299a-5p inhibitor before treatment with 5 μM ELA. (22) H/R + ELA + U0126 group: BMSCs pretreated with 10 μM U0126 (ERK pathway inhibitor; Med Chem Express, United States) for 6 h before 5 μM ELA. Groups (12)–(22) were cultured in H/R-NRVMs medium for 12 h. Groups (13)–(22) were treated with 5 μM ELA for 24 h.

Treatments (1)–(8) involved various siRNA applications to BMSCs. The comparisons in (9) and (10) were pairwise, with specific statistical methods detailed in section 2.18(hereafter the same). Comparisons in (11)–(13) included multiple groups, and subsequent comparisons in (11)–(15) concentrated on pairwise comparisons following Exo70 knockdown and its effects on phenotypes. Similarly, (11)–(13) and (16)–(19) investigated the impact of miR-299a-5p modulation on downstream factors and phenotypes. Comparisons in (11)–(13) and (20) evaluated the effects of ERK pathway inhibition, and (11)–(13), (21), and (22) examined the consequences of APJ receptor inhibition on downstream factors and phenotypes.

### 2.10 Transwell assays

Transwell assays using Transwell chambers with an 8-μm pore size (Corning, United States) were conducted to examine the targeting efficiency of BMSCs to injured cardiomyocytes *in vitro*. BMSCs were serum starved for 12 h before transwell assays. NRVMs, subjected to H/R conditions (12 h of hypoxia followed by 12 h of reoxygenation in low-glucose DMEM with 10% serum) were placed in the lower chamber (6-well plates), while BMSCs (5 × 10^5^ cells/well) were seeded in the upper chamber. After incubation for 12 h at 37°C, non-migrated cells in the upper chamber were removed with cotton swabs, and migrated cells were fixed with 4% paraformaldehyde for 30 min. After drying, the upper chambers were stained with 0.1% crystal violet dyes for 20 min at room temperature. The number of migrated cells was observed under an inverted microscope and counted using ImageJ software (ImageJ 1.53t, United States).

### 2.11 Scratch assay

BMSCs migration was measured using an *in vitro* scratch wound assay. Briefly, BMSCs were seeded in a 6-well plate to achieve 90% confluency. The culture medium was then removed, and a wound was made in the middle of the well using a 200 µL micropipette tip. Then, each well was washed three times with PBS to eliminate cell debris. NRVMs medium and H/R-NRVMs medium were separately added to the different groups. Wound closure was photographed with an inverted microscope at 0 and 12 h post-wounding. A percentage of wound closure was calculated using ImageJ software (ImageJ 1.53t, United States) by the following formula: (original wound area-remaining wound area)/original wound area ×100%.

### 2.12 IBIDI cell migration studies and live video microscopy

To dynamically observe the cells’ motility trajectory, BMSCs chemotaxis experiments were performed on the µ-Slide Chemotaxis chamber (Ibidi, #80326, Germany) treated at the bottom of the ibiTreat according to the protocol provided by the manufacturer. Cultures containing 2 × 10^6^ cells were placed in the central channel of μ-slide chambers. After 6 h of incubation, a chemotaxis gradient was established by adding NRVMs culture to the left medium reservoir and H/R-NRVMs culture to the right medium reservoir. The movement of BMSCs in the central channel was recorded over time using a time-lapse image system under an inverted confocal microscope (Nikon Ti2-E, Japan), with temperature and CO_2_ concentration controlled by a companion live cell workstation (TOKAI HIT, Japan). Light field images were acquired every 10 min for 20 h, and the trajectory of at least 30 cells within the observation area of each image sequence was tracked manually using the ImageJ Manual Tracking plug-in (ImageJ 1.53t, United States). To evaluate the efficacy of ELA on BMSCs, we determined the migration efficiency of BMSCs by a combination of evaluation metrics such as the forward migration index (FMI), migration velocity and distance after multiple experiments.

### 2.13 Transcriptome sequencing and analysis

Transcriptome sequencing and analysis were conducted by Shanghai GeneChem Co., Ltd. (Shanghai, China). Sequencing libraries were built using NEBNext^®^ UltraTM RNA Library Prep Kit for Illumina^®^ (NEB, United States) following the manufacturer’s recommendations. The library preparations were sequenced on the Illumina Nova6000 platform to generate 150 bp paired-end reads. All the bioinformatic analyses were based on the clean data with high quality.

### 2.14 Plasmid transfection and luciferase assay

MiR-299a-5p potentially targeting the 3′-UTR of the Exo70 gene was predicted by the TargetScan (http://www.targetscan.org/) and miRWalk (http://mirwalk.umm.uni-heidelberg.de/). 293T cells were seeded in 24-well plates (1 × 10^5^ cells/well) and cultured until the cell confluency reached 60%. Plasmid names were listed in Table 2. Plasmid transfections were performed by using X-tremeGENE HP DNA transfection reagent (Roche, Switzerland) according to the instruction manual. The cells were harvested 48 h after transfection and measured using the Dual-Luciferase Reporter Assay System (Promega, United States).

**TABLE 2:** Primer sequences of RT-qPCR.

Primer	Sequences (5′-3′)
Exo70	F: CAG​CTA​TTA​CCA​CGT​AGC​CAG​C
	R: ATC​TGG​GCT​GTT​GTC​CTG​AAA​G
GAPDH	F: CCT​CGT​CTC​ATA​GAC​AAG​ATG​GT
	R: GGG​TAG​AGT​CAT​ACT​GGA​ACA​TG
miR-299a-5p	F: GCC​GAG​TGG​TTT​ACC​GTC​C
	R: GTC​GTA​TCC​AGT​gCA​GGG​TCC​G
	RT: GTCGTATCCAGTGCAGGGTCCGAGGTATT CGCACTGGATACGACATGTATGT
U6	F: CCTGCTTCGGCAGCACA
	R: AAC​GCC​TCA​CGA​ATT​TGC​GT

### 2.15 Reverse transcription-quantitative polymerase chain reaction (RT-qPCR)

Total RNA was extracted from BMSCs using Trizol reagent (Sigma, United States) and quantified using a NanoDrop spectrophotometer (ThermoFisher Scientific). The primer sequences were listed in Table 2. The cDNA was then synthesized by reverse transcription kit (RR047A, TaKaRa, Japan). RT-qPCR was performed on LightCycler^®^ 480 real-time PCR Platform (Roch e, Switzerland) using TB Green^®^ Premix Ex Taq TM (RR820A, TaKaRa, Japan). The relative miRNA levels were normalized to U6 small nuclear RNA levels. The relative RNA level was calculated using the 2^−ΔΔCT^ method. Each group had three replicates, and the experiment was conducted independently three times.

### 2.16 Western blotting

Total cellular proteins were extracted from BMSCs using RIPA lysis buffer (RIPA, Beyotime, China) supplemented with protease and phosphatase inhibitor cocktail (CWBIO, China) for 30 min on ice. Protein concentration was determined using the bicinchoninic acid (BCA) protein assay kit (CWBIO, China). Protein samples were mixed with 5 × SDS-PAGE sample loading buffer and heated at 70°C for 10 min. Proteins were separated by 10% SDS-PAGE and transferred to a 0.2 μm PVDF membrane (Millipore, United States). Membranes were blocked in 5% skimmed milk for 1 h and incubated overnight at 4°C with primary antibodies [Exo70 (1:1000; #12014-1-AP; Proteintech, United States), phospho-p44/42 MAPK (p-ERK1/2) (1:2000; #4370; Cell Signaling Technology, United States), p44/42 MAPK (ERK1/2) (1:1000; #4695; Cell Signaling Technology, United States) and GAPDH (1:1000; #2118; Cell Signaling Technology, United States)]. After overnight incubated, membranes were washed three times with TBST for 10 min each time and incubated with HRP-linked secondary antibody: anti-rabbit IgG (1:3,000, Cell Signaling Technology, United States). Protein bands were detected using chemiluminescence reagents and visualized with ChemiDoc™ Touch Imaging System (Bio-Rad, United States).

### 2.17 F-actin staining

Immunofluorescence staining was used to determine polymerized F-actin using the F-actin staining kit-Green Fluorescence-Cytopainter (#ab112125, Abcam, US). BMSCs were fixed with 4% paraformaldehyde for 30 min followed by permeabilization with 0.1% Triton X-100 for 5 min at room temperature. After washing with PBS for three times, the Green Fluorescent Phalloidin Conjugate working solution was added into fixed BMSCs for 20 min at room temperature in the dark. Excess dye was washed with PBS before nuclei were stained with 4′, 6-diamidino-2-phenylindole (DAPI, beyotime, China) for 10 min at room temperature in the dark. Fluorescence images were taken using a confocal laser microscope (LSM880, Zeiss, Germany). The fluorescence intensity was quantified using ImageJ software (ImageJ 1.53t, United States), followed by statistical analysis. An increased fluorescence intensity signifies an enhanced ability for F-actin polymerization.

### 2.18 Statistical analysis

**FIGURE 1:**
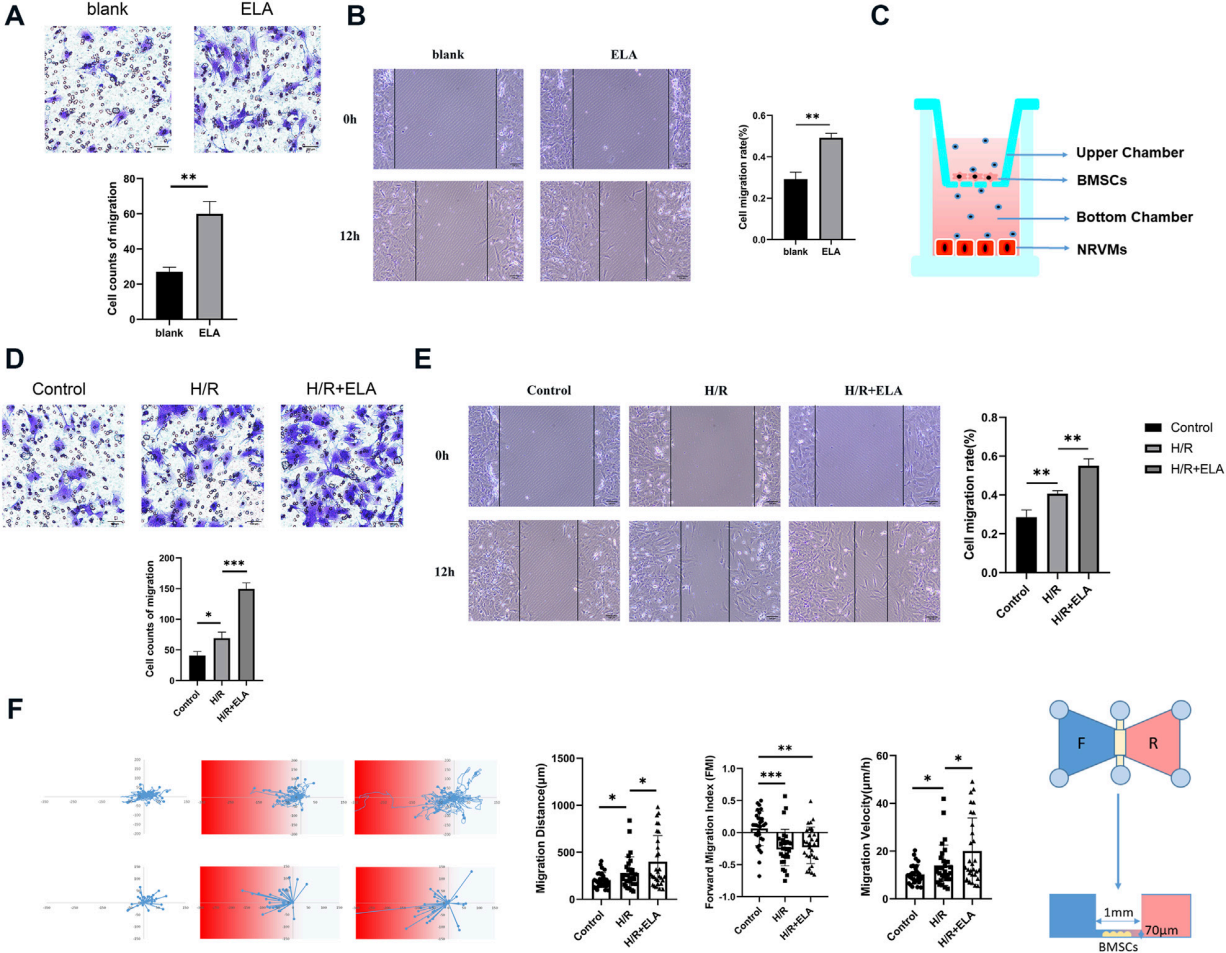
ELA pretreatment promoted BMSCs migration and homing. **(A)** Transwell migration of BMSCs in the blank and ELA pretreatment groups. **(B)** Wound closure of BMSCs in the blank and ELA pretreatment groups. **(C)** A transwell model of migration of BMSCs to damaged cardiomyocytes. **(D)** Transwell cell migration in different model treatment groups. **(E)** The wound closure of BMSCs in different model treatment groups. **(F)** The migration distance, velocity and forward migration index of individual cells based on the real-time migration trajectory of BMSCs by Ibidi migration model in different groups. The white/blue zone: the NRVMs culture; The red zone: the H/R-NRVMs culture; n = 30. The other experiments above were repeated three times (n = 3). Scale bar = 100 μm. *p < 0.05, **p < 0.01, ***p < 0.001. ELA: ELABELA; BMSCs: Bone marrow mesenchymal stem cells; H/R: Hypoxia reoxygenation.

**FIGURE 2:**
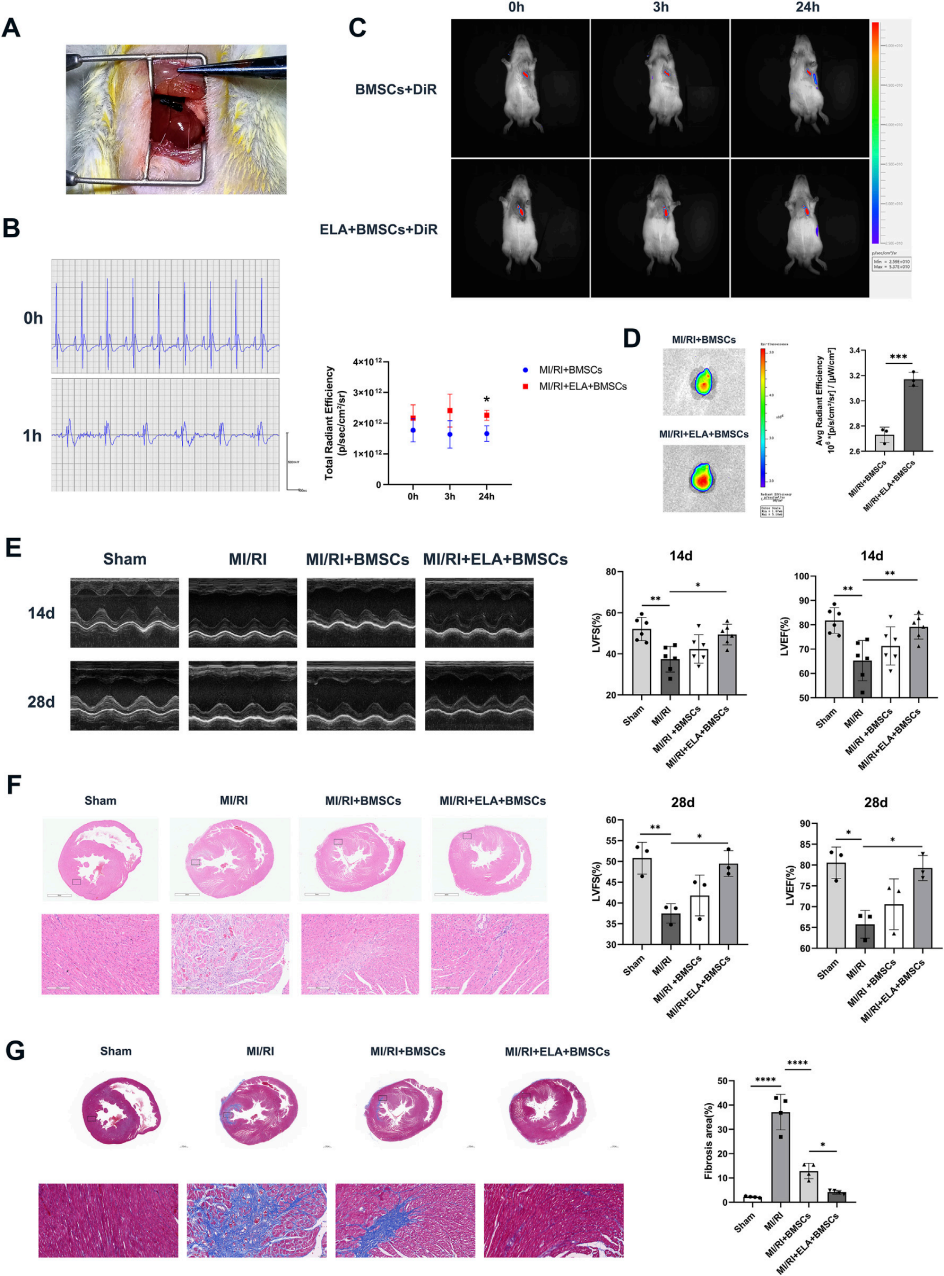
ELA pretreatment enhanced BMSC-mediated repair of myocardial injury. **(A)** Surgical view following ligation of the left anterior descending branch of the coronary artery. **(B)** Preoperative and 1 h postoperative electrocardiograms (ECGs) of rats. **(C)** *In vivo* imaging of cells stained with DiR in various groups, along with a comparative analysis of total radiant efficiency (n = 3). **(D)** DiR average fluorescence imaging of the heart at 28 days after BMSCs injection via rat tail vein (n = 3). **(E)** An analysis of echocardiograms, specifically LVFS and LVEF results, for different treatment groups at 14 days (n = 6) and 28 days (n = 3). **(F)** Panoramic and localized images of HE staining in different treatment groups. **(G)** Panoramic and localized images of Masson staining in different treatment groups, along with analysis of the fibrosis rate (n = 4). Scale bar = 200 μm. *p < 0.05, **p < 0.01, ****p < 0.0001. ELA: ELABELA; BMSCs: Bone marrow mesenchymal stem cells; MI/RI: myocardial ischemia-reperfusion injury; LVEF: Left ventricular ejection fraction; LVFS: Left ventricular fractional shortening.

**FIGURE 3:**
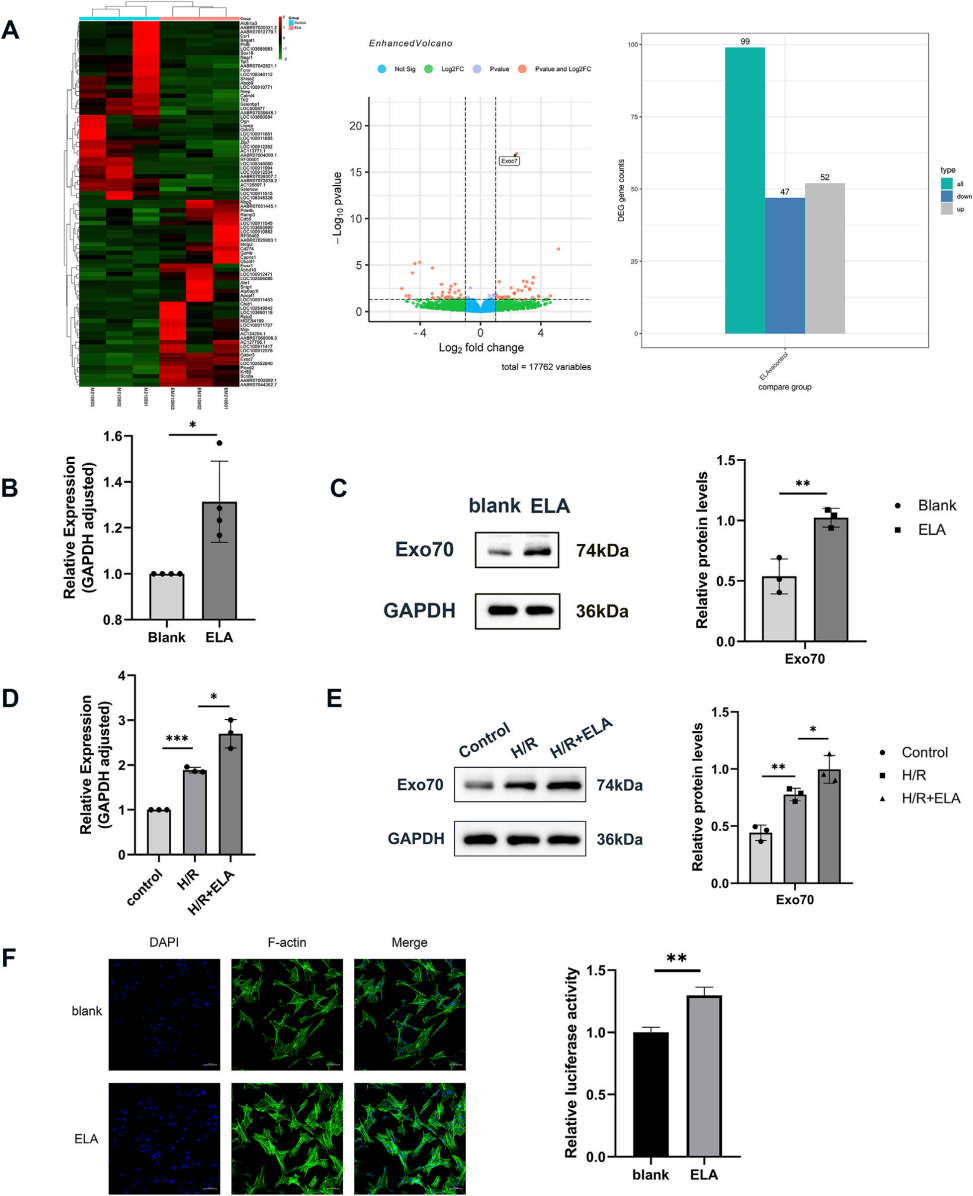
Transcriptome screening and validation of ELA to promote cell migration. **(A)** Heat map and volcano plot of 99 differential genes screened by transcriptome sequencing. **(B)** Relative expression of Exo70 in BMSCs in blank group and ELA pretreatment group by qPCR. **(C)** Expression of Exo70 in BMSCs in the blank group and ELA pretreatment group by Western blotting. **(D)** Relative expression of Exo70 in BMSCs in different model treatment groups by qPCR. **(E)** Expression of Exo70 in BMSCs in different model treatment groups by Western blotting. **(F)** Polymerization of F-actin in two different groups. All experiments were repeated at least three times (n ≥ 3). Scale bar = 100 μm. *p < 0.05, **p < 0.01. GAPDH was used as an internal reference. ELA: ELABELA; BMSCs: Bone marrow mesenchymal stem cells; H/R: Hypoxia reoxygenation. Exo70: Exocyst complex component 7.

**FIGURE 4:**
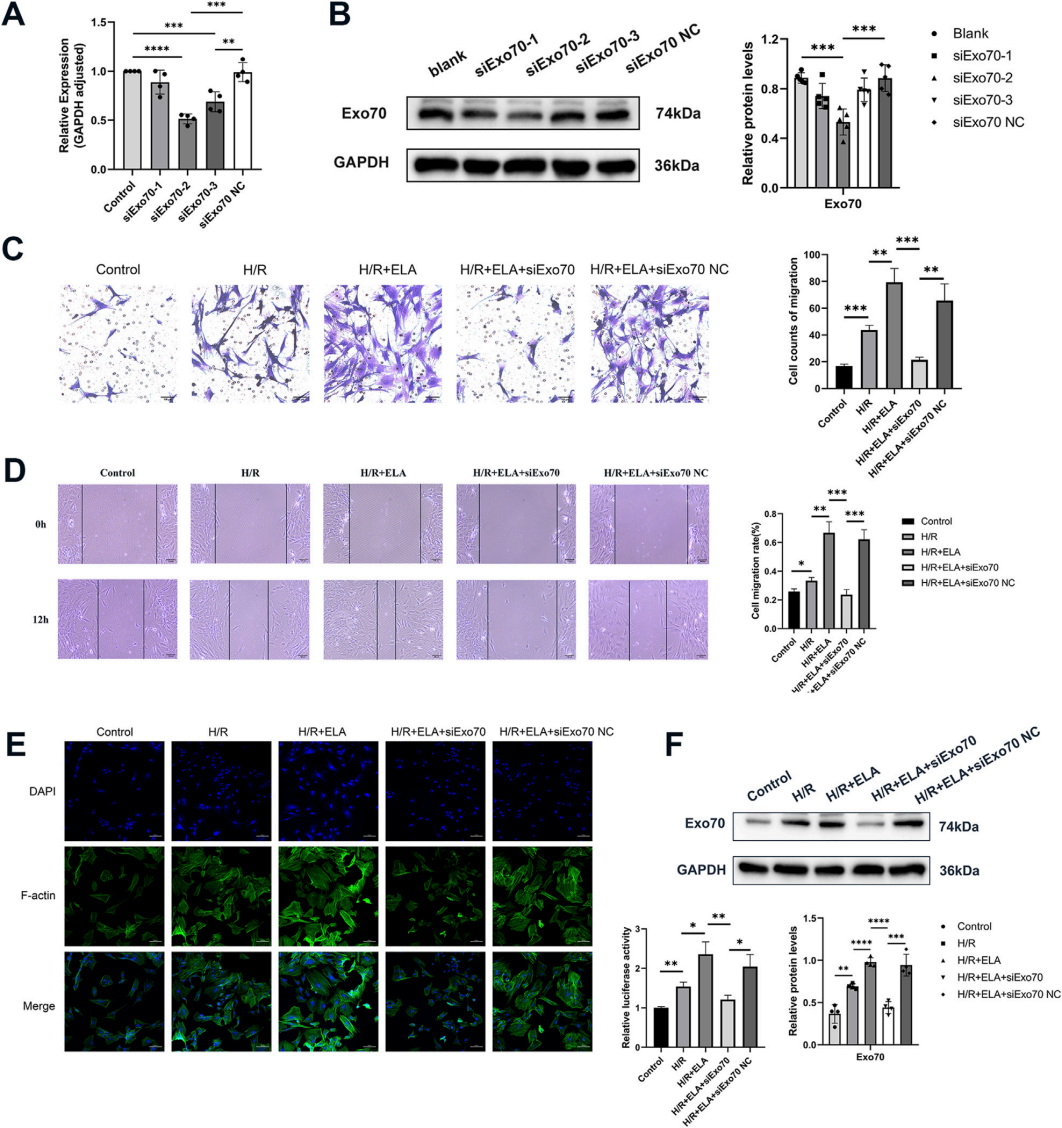
ELA promoted BMSC migration by modulating Exo70. **(A)** The selection of siExo70 by qPCR. **(B)** The selection of siExo70 by Western blotting. **(C)** Transwell migration of BMSCs after silencing of Exo70. **(D)** Scratch healing of BMSCs after silencing of Exo70. **(E)** Polymerization of F-actin of BMSCs after silencing of Exo70. **(F)** Expression of Exo70 in BMSCs in different model treatment groups by Western blotting. All experiments were repeated at least three times (n ≥ 3). Scale bar = 100 μm. *p < 0.05, **p < 0.01, ***p < 0.001, ****p < 0.0001. GAPDH was used as an internal reference. ELA: ELABELA; BMSCs: Bone marrow mesenchymal stem cells; H/R: Hypoxia reoxygenation. Exo70: Exocyst complex component 7. siExo70: Exo70 small interfering RNA.

**FIGURE 5:**
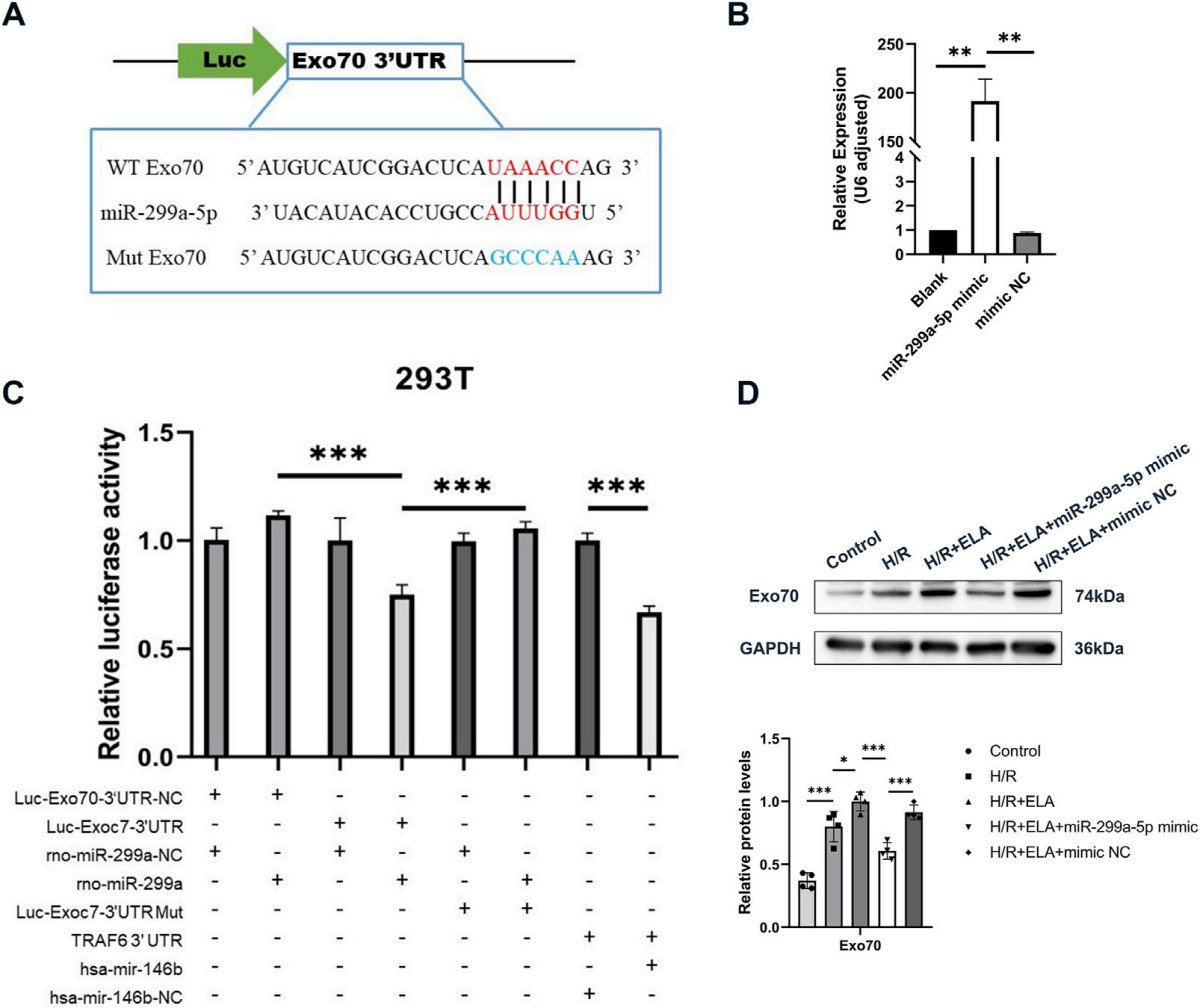
Targeted relationship between Exo70 and miR-299a-5p. **(A)** Predictive map of the possible targeting relationship of Exo70 and miR-299a-5p. **(B)** Relative expression of miR-299a-5p in BMSCs in different treatment groups by qPCR. **(C)** Relative luciferase activity of different treatment groups in 293T. **(D)** Expression of Exo70 protein after overexpression of miR-299a-5p. All experiments were repeated at least three times (n ≥ 3). *p < 0.05, **p < 0.01, ***p < 0.001. All miRNA expression was normalized to U6 small nuclear RNA. GAPDH serves as an internal reference protein. ELA: ELABELA; BMSCs: Bone marrow mesenchymal stem cells; H/R: Hypoxia reoxygenation; Exo70: Exocyst complex component 7; siExo70: Exo70 small interfering RNA; miR: MicroRNA.

**FIGURE 6:**
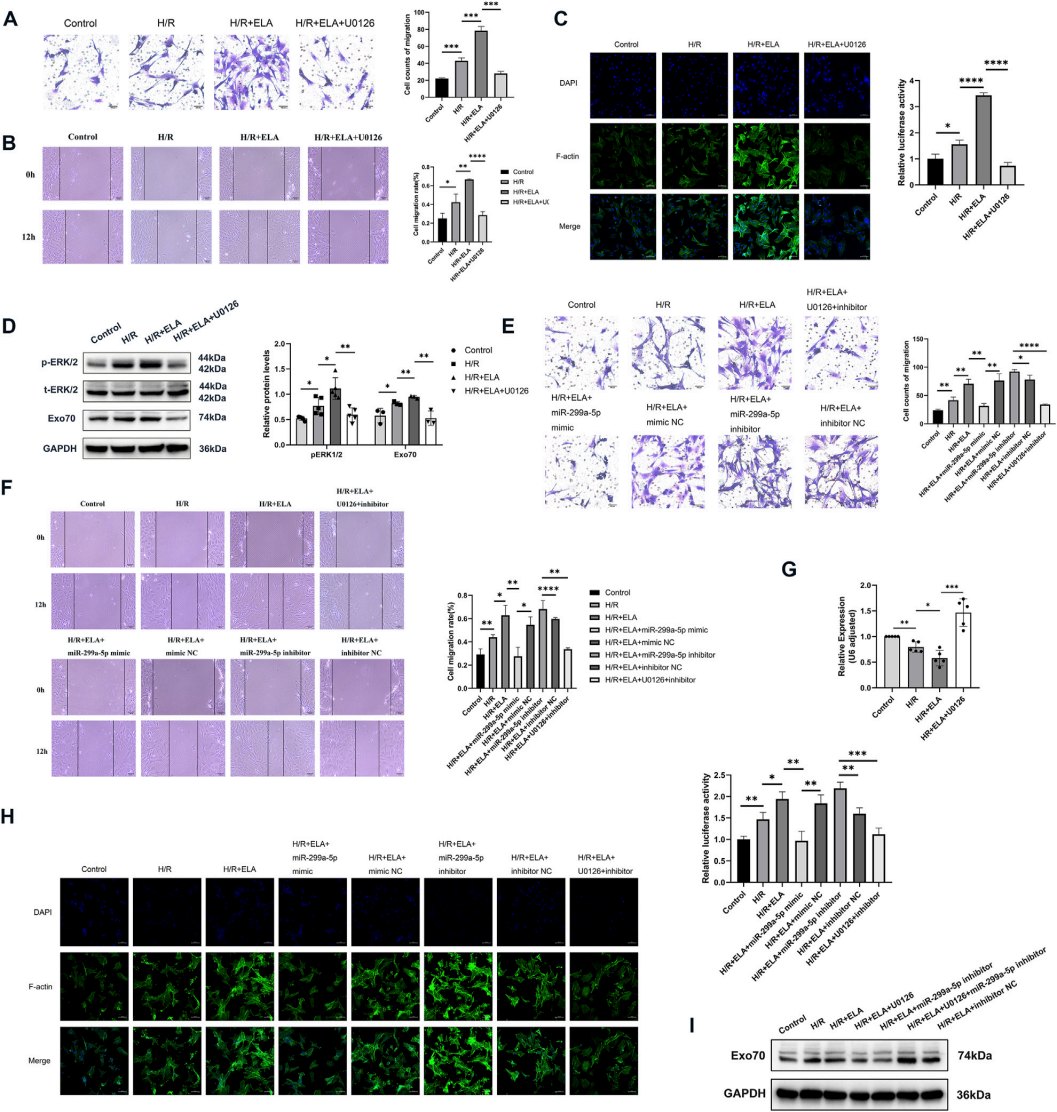
ELA regulated miR-299a-5p and Exo70 expression through the ERK1/2 pathway. **(A)** Transwell migration of BMSCs after using ERK pathway inhibitor (U0126). **(B)** Scratch healing of BMSCs after using U0126. **(C)** Polymerization of F-actin of BMSCs after using U0126. **(D)** The expression of different proteins after using U0126. **(E)** Transwell migration of BMSCs in different treatment groups. **(F)** Scratch healing of BMSCs in different treatment groups. **(G)** Relative expression of miR-299a-5p in different treatment groups by qPCR. **(H)** Polymerization of F-actin of BMSCs in different treatment groups. **(I)** The expression of Exo70 in different treatment groups by Western blotting. All experiments were repeated at least three times (n ≥ 3). *p < 0.05, **p < 0.01, ***p < 0.001, ****p < 0.0001. All miRNA expression was normalized to U6 small nuclear RNA. GAPDH serves as an internal reference protein. ELA: ELABELA; BMSCs: Bone marrow mesenchymal stem cells; H/R: Hypoxia reoxygenation; Exo70: Exocyst complex component 7; siExo70: Exo70 small interfering RNA; p-ERK1/2: Phosphorylated extracellular regulated protein kinases1/2; t-ERK1/2: Total extracellular regulated protein kinases 1/2; miR: MicroRNA.

**FIGURE 7:**
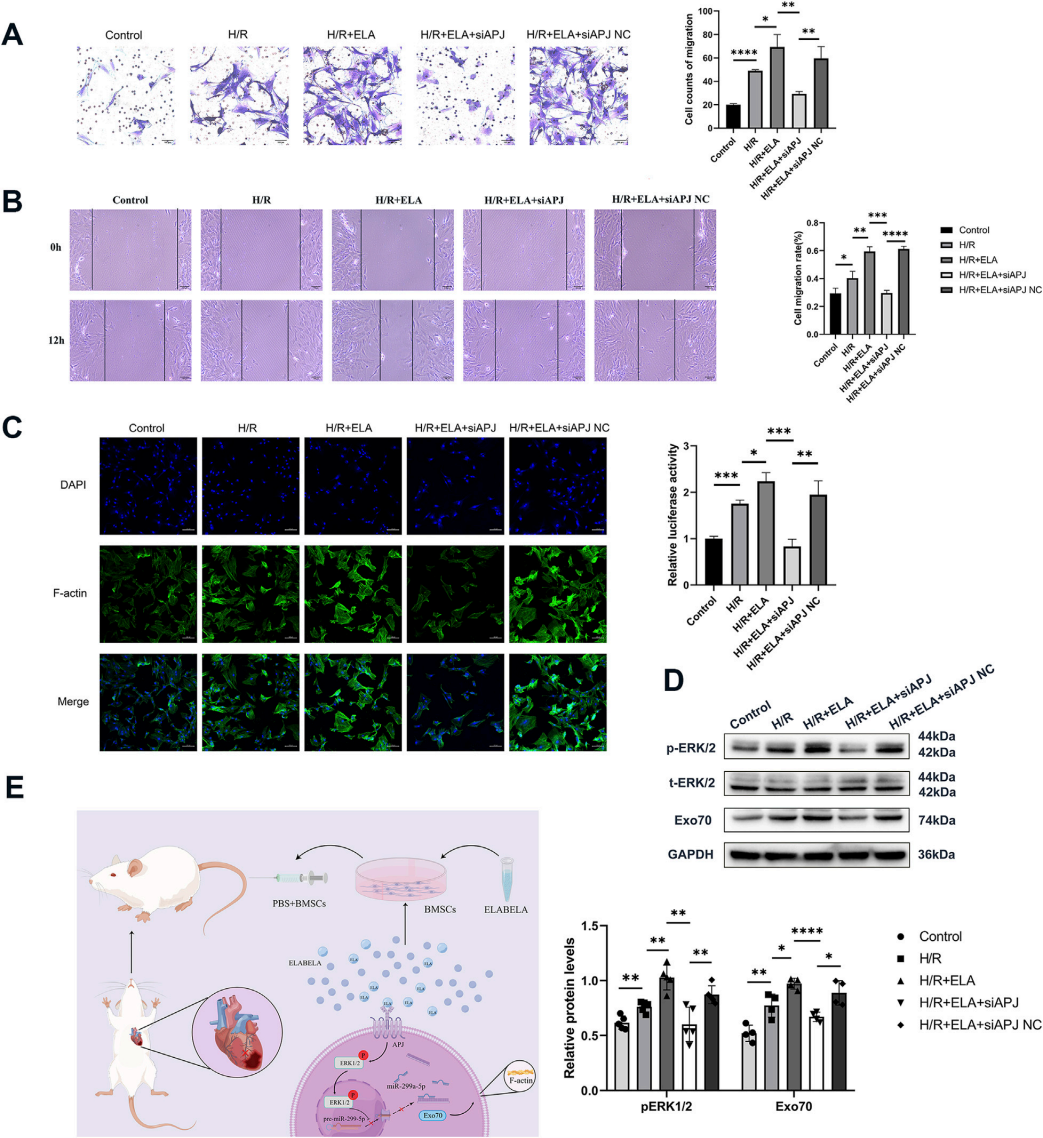
ELA regulated miR-299a-5p and Exo70 expression through the APJ receptor-activated ERK1/2 pathway. **(A)** Transwell migration of BMSCs after silencing of APJ. **(B)** Scratch healing of BMSCs after silencing of APJ. **(C)** Polymerization of F-actin of BMSCs after silencing of APJ. **(D)** Expression of different proteins after silencing of APJ. **(E)** Diagram of possible molecular mechanisms by which ELA promoted the migration of BMSCs. All experiments were repeated at least three times (n ≥ 3). *p < 0.05, **p < 0.01, ***p < 0.001,****p < 0.0001. All miRNA expression was normalized to U6 small nuclear RNA. GAPDH serves as an internal reference protein. ELA: ELABELA; BMSCs: Bone marrow mesenchymal stem cells; H/R: Hypoxia reoxygenation; Exo70: Exocyst complex component 7; siExo70: Exo70 small interfering RNA; p-ERK1/2: Phosphorylated extracellular regulated protein kinases1/2; t-ERK1/2: Total extracellular regulated protein kinases 1/2; APJ: Apelin receptor; siAPJ: APJ small interfering RNA; miR: MicroRNA.

Data were plotted using GraphPad Prism 8 software (GraphPad, United States). Statistical significance was assessed based on the results of at least three independent experiments. An analysis that involved comparing two groups was conducted using Student's t-test. Comparisons among multiple groups were assessed using one-way analysis of variance (ANOVA) with a Bonferroni post hoc test. Pairwise comparisons between groups (e.g., the blank group vs. the ELA group, the Control group vs. the H/R group, and the H/R group vs. the H/R + ELA group, etc.) were shown in Figures 1–7. The data were expressed as Mean ± SD. Statistical significance was determined by P < 0.05.

## 3 Results

### 3.1 ELA pretreatment promoted BMSCs migration and homing under H/R conditions

Cell migration analysis showed that the ELA group exhibited a significantly higher migration ability than the blank group (p < 0.01) (Figures 1A, B). Simultaneously, in the scratch model and transwell migration template as showed in Figure 1C, the migration rate of the H/R + ELA group was notably higher than that of the H/R group (p < 0.05), while the migration rate of the control group was lower than that of the H/R group (p < 0.05) (Figures 1D, E). Dynamic tracking of BMSCs motility was conducted using the Ibidi Chemotaxis and Migration Tool, which visualized individual tracks. Both the H/R and H/R + ELA groups showed enhanced chemotaxis capacity towards the H/R-NRVMs medium (p < 0.05). The H/R + ELA group showed greater migration distance and faster velocity compared to the H/R group (p < 0.05) (Figure 1F).

### 3.2 ELA pretreatment enhanced BMSCs-mediated myocardial repair

Following the ligation of the left anterior descending branch of the coronary artery, localized myocardial ischemia was visually apparent below the ligature line, with a pale appearance (Figure 2A). One hour after reperfusion, ECG demonstrated ST segment elevation in the precordial leads, confirming the successful establishment of myocardial infarction/reperfusion injury (MI/RI) (Figure 2B). At this stage, DiR-labeled BMSCs and ELA + BMSCs were intravenously administered through the tail vein. Immediate *in vivo* imaging of rats indicated that DiR fluorescence primarily localized in the heart, with no statistically significant difference observed between the two groups (with and without ELA pre-treatment) immediately and 3 h after cell injection. However, after 24 h, the ELA pre-treatment group exhibited significantly stronger fluorescence intensity in the heart region compared to the non-pretreated group (p < 0.05, Figure 2C). At 28 days post-injection, fluorescence imaging of the heart revealed that the average radiant efficiency of the ELA pre-treatment group was significantly higher than that of the MI/RI + BMSCs group (p < 0.001, Figure 2D). These findings suggested that ELA pre-treatment enhanced BMSCs migration and homing to the injured myocardium.

Cardiac function evaluation at 14 and 28 days post-surgery revealed significant reductions in LVFS (%) and LVEF (%) values in the MI/RI group compared to the sham group (p < 0.05). While the MI/RI + BMSCs group exhibited increased LVFS (%) and LVEF values, the differences were not statistically significant (p > 0.05). In contrast, significant increases in LVFS (%) and LVEF values were observed in the MI/RI + ELA + BMSCs group compared to the MI/RI group, suggesting the beneficial effects of ELA pretreatment in improving myocardial function (Figure 2E). Histological examination through HE staining (Figure 2F) revealed that the myocardial structure in the sham group remained predominantly intact, whereas myocardial damage was observed in the MI/RI, MI/RI + BMSCs, and MI/RI + ELA + BMSCs groups. Within the infarcted area, the MI/RI group retained some myocardial tissue with a relatively ample presence of blood vessels, yet notable myocardial fibrosis was evident. The fibrotic region was distinguished by lax and disordered fibrous regions, accompanied by extensive infiltration of inflammatory cells. In contrast, the MI/RI + BMSCs group exhibited a high density of myocardial scar formation within the infarcted region, indicative of the advanced stage of myocardial infarction repair. Conversely, the MI/RI + ELA + BMSCs group displayed a reduced scar area in comparison to the MI/RI + BMSCs group, suggesting a notable enhanced myocardial repair. Masson’s staining confirmed that the fibrosis area was significantly lower in the MI/RI + ELA + BMSCs group compared to the MI/RI + BMSCs group (p < 0.05, Figure 2G).

### 3.3 Transcriptome screening and validation of ELA in promoting cell migration

Transcriptome analysis identified 99 differentially expressed genes (Figure 3A), of which 47 were downregulated and 52 were upregulated. Among the top 20 upregulated genes, Exo70 was highly expressed in BMSCs and implicated in cell migration. Exo70 expression was validated at both the RNA and protein levels, showing a significant increase in BMSCs pretreated with ELA (p < 0.05). Additionally, Exo70 expression was increased in the H/R group compared to the control group. Exo70 expression was higher in the H/R + ELA group than in the H/R group (p < 0.05) (Figures 3B–E). The above results showed that ELA played a supranormal role in enhancing cell migration under a H/R environment. The F-actin immunofluorescence assay further revealed that the immunofluorescence intensity was enhanced in the H/R + ELA group, indicating that ELA could promote actin polymerization (Figure 3F), thus promoting cell migration.

### 3.4 ELA promoted BMSCs migration by modulating Exo70

To confirm the role of Exo70 in BMSCs migration, Exo70 was silenced using siRNA. qPCR and WB showed that the second pair of siExo70 efficiently silenced Exo70 expression (p < 0.05, Figures 4A, B, F). In the H/R condition, the migration rate of the ELA-treated group was higher than that of the ELA-free group but silencing Exo70 with siExo70 significantly reduced migration in the H/R + ELA group (P < 0.05, Figures 4C–E). The above results suggest that siExo70 reversed the effect of ELA in promoting MSC migration and that Exo70 is an essential part in the process of ELA promoting BMSCs migration.

### 3.5 Exo70 as a target of miR-299a-5p

Bioinformatics prediction indicated a potential interaction between Exo70 and miR-299a-5p (Figure 5A). Transfection of miR-299a-5p mimic significantly increased miR-299a-5p expression (p < 0.01, Figure 5B). A dual luciferase reporter was used to detect microRNAs regulating the expression of target genes mainly through its binding to the 3′end untranslated region (3′UTR) of the target gene mRNA, resulting in degradation of the target mRNA or inhibition of protein synthesis. This implied that comparisons between Firefly/Renilla luminescence fold, calibrated to eliminate differences and re-quantified in the presence of identical miR-299a plasmids, were used to determine whether target gene Exo70 3′UTR null, target gene Exo70 3′UTR, target gene Exo70 3′UTR mutant plasmids bound to miR-299a and inhibited luciferase expression. The results showed that compared to the positive reference miRNA NC group, there was a significant decrease (p < 0.001) in the expression of relative luciferase in the positive reference miRNA group, indicating that there was no problem with the whole transfection assay system. miR-299a-5p bound to the 3′UTR of Exo70 and inhibited its expression, as evidenced by significantly reduced luciferase activity in the Luc-Exo70-3′UTR + miR-299a group (p < 0.001, Figure 5C). At the protein level, the expression of Exo70 was significantly decreased in the H/R + ELA group after overexpression of miR-299a-5p (p < 0.001, Figure 5D). In conclusion, all the above results demonstrated that Exo70 was a direct target of miR-299a-5p.

### 3.6 ELA regulated miR-299a-5p and Exo70 via the ERK1/2 pathway

To further explore the mechanism of migration promotion by ELA in BMSCs, we examined migration-related kinases. We found that the phosphorylation level of ERK1/2 was increased after treatment with ELA under H/R conditions. The ERK pathway inhibitor U0126 significantly reduced ERK1/2 phosphorylation (Figure 6D) and reversed the pro-migration effect of ELA (Figures 6A, B), as well as affecting F-actin polymerization (Figure 6C). These results suggest that the ERK1/2 pathway was involved in ELA-induced BMSCs migration. Further analysis showed that ERK1/2 phosphorylation modulated miR-299a-5p and Exo70 expression, as both miR-299a-5p levels and Exo70 expression were affected by ERK1/2 inhibition by U0126 (Figures 6D, G). Moreover, combining U0126 with miR-299a-5p Inhibitor did not significantly improve migration (p > 0.05) or F-actin polymerization (Figures 6E, F, H, I), indicating that the ERK1/2 pathway mediates ELA-induced migration through miR-299a-5p and Exo70 regulation.

### 3.7 ELA regulated miR-299a-5p and Exo70 expression through APJ receptor-activated ERK1/2 pathway

Our previous studies confirmed that APJ is an important receptor regulating BMSCs survival, and the suppression of APJ by siRNA has been demonstrated in our earlier work (Fu et al., 2020). To further explore whether ELA promoted F-actin polymerization and thus affected migration through the APJ receptor, we performed APJ gene silencing and found that siAPJ inhibited BMSCs migration efficiency (Figures 7A, B) and affected F-actin polymerization (Figure 7C). Western blot analysis revealed that silencing APJ suppressed ERK1/2 phosphorylation and reduced Ex070 expression (P < 0.05, Figure 7D). Taken together, our data suggest that ELA regulated miR-299a-5p and Exo70 expression through APJ receptor activation of the ERK1/2 pathway, as depicted in the proposed mechanistic model (Figure 7E).

## 4 Discussion

In this study, we demonstrated that ELA promotes F-actin polymerization in BMSCs, which facilitates their migration and homing to damaged cardiomyocytes. The underlying mechanism of ELA-enhanced migration was confirmed by transcriptome sequencing, bioinformatics analysis, and experimental validations. We identified that the activation of ERK1/2 phosphorylation, along with thedown-regulation of miR-299a-5p and upregulation of Exo70, played pivotal roles in this process. This study offer novel insights into the molecular mechanisms involved in the transplantation of BMSCs for AMI and provide a more reliable theoretical and experimental basis for exploring ways to enhance the efficacy of BMSCs transplantation.

Currently, most clinical and preclinical research evidence supports the use of MSCs, especially bone marrow-derived MSCs, as a safe and promising biologic therapy in the cellular treatment of AMI, which enhances not only cardiac angiogenesis and significantly improves cardiac function after injury but also has myocardial regenerative potential (Szydlak, 2023; Thavapalachandran et al., 2021). Systematic evaluations and *in vivo* safety trials have demonstrated that BMSCs transplantation is generally safe, with minimal toxic side effects. However, its therapeutic potential in terms of functional recovery has shown mixed results (Gao et al., 2022; Yu et al., 2022). Autologous MSCs transplantation after MI was not found to significantly promote the recovery of LV function and myocardial viability (Zhang R. et al., 2021), or the improvement was minimal. Meta-analyses in some literature showed only a 2.62% (Yu et al., 2022) or 3.78% (Attar et al., 2021) increase in LVEF after transplantation. This limited benefit could be attributed to the inadequate migration and homing of MSCs to the infarcted tissue, which is further complicated by the hypoxic-ischemic environment of AMI. The success of BMSCs transplantation largely depends on the migration and homing efficiency of the cells to the damaged myocardium (Szydlak, 2021). Unfortunately, BMSCs face significant challenges in achieving optimal migration and survival rates solely due to environmental factors and inherent characteristics. To address these challenges, researchers tried to promote the migration of BMSCs with natural botanicals or chemically synthesized drugs (Han et al., 2019; Li et al., 2021; Lu et al., 2023; Wang et al., 2024). Despite these efforts, no single treatment has yet achieved a substantial improvement in graft survival or migration rates while ensuring patient safety. In our study, ELA showed no adverse effects on the APJ receptor, as confirmed by previous cardiovascular monitoring and safety reports (Gourdy et al., 2018). Our findings indicate that ELA pre-treatment significantly enhances BMSCs migration both *in vitro* and *in vivo*, as evidenced by transwell assays, scratch assays, and an MI/RI rat model. Additionally, ELA-pretreated BMSCs demonstrated significant efficacy in the repair of myocardial injury and preservation of cardiac function.

To further investigate the mechanism by which ELA promotes the migration of BMSCs, we performed transcriptome sequencing and bioinformatics analysis to screen the top 20 differentially expressed genes and found significant differences in the presence of the Exo70 gene between the two groups (p < 0.05). Exo70 is a key component of an octameric protein complex that facilitates the association of the Arp2/3 complex with the Wiskott-Aldrich syndrome protein family verproline-homologous protein-2 (WAVE2), thereby influencing F-actin polymerization and playing a crucial role in the formation of lamellar pseudopods and the maintenance of cell migration orientation persistence (Liu et al., 2012; Zhu et al., 2019; Kullmann et al., 2020), While Exo70 has been implicated in tumor cell migration, its role in stem cell therapy has not been fully explored. Our study found that ELA upregulates Exo70 in BMSCs, and silencing Exo70 with siRNA significantly impairs BMSCs migration and reduces F-actin polymerization indicating that Exo70 is a key mediator in ELA-induced BMSCs migration. This suggests that ELA pre-treatment may promote the migration of MSCs to damaged cardiomyocytes by upregulating Exo70.

MicroRNAs (miRNAs) are small endogenous RNAs consisting of 18–23 nucleotides that regulate the expression of target genes. Combined with bioinformatics analysis (TargetScan, http://www.targetscan.org), miR-299-5p (miR-299a-5p) was predicted to bind to the 3′-UTR of Exo70 in this study. MiR-299-5p, one of many miRNA molecules, played an essential biological function in inflammation and tumor development, participating in critical cellular processes such as epithelial-mesenchymal transition, proliferation, and migration (Fateh et al., 2017). (Formosa et al., 2014)analyzed the GSE21032 dataset available on the GEO website and concluded that miR-299-5p showed a significant and statistically significant trend of downregulated expression from normal tissue to localized tumors to metastatic tumors. Another study suggested that miR-299-5p was closely associated with the migration and invasion of papillary thyroid cancer (Wang et al., 2018). Although the role of miR-299a-5p in MSC migration is less well-studied, one study indicated that MSCs could secrete miR-299a-5p, which influences osteogenic differentiation (Kaneda-Ikeda et al., 2020). In our study, we observed a significant downregulation of miR-299a-5p in ELA-pretreated BMSCs, inhibition of cell migration function after overexpression of miR-299a-5p, and an impact on F-actin polymerization. The double luciferase assay verified that Exo70 was a direct target gene of miR-299a-5p. MiR-299a-5p could bind to Exo70 and inhibit Exo70 expression, thus affecting its ability to promote the polymerization of F-actin. These findings indicate that ELA promotes BMSCs migration to injured cardiomyocytes by down-regulating miR-299a-5p, leading to an upregulation of Exo70 and subsequent F-actin polymerization.

The intermediate mechanism underlying ELA-induced downregulation of miR-299a-5p attracted our interest. Activation of the ERK1/2 signaling pathway has been shown to regulate miRNA expression and promote cell migration (Sun et al., 2016; Szokodi et al., 2008). Our previous study demonstrated that ELA could significantly attenuate ischemia-reperfusion-induced apoptosis and improve the survival rate of BMSCs by regulating ERK1/2 phosphorylation (Fu et al., 2020). However, it has not been reported whether the mechanism by which ELA promotes migration and homing of BMSCs is achieved through the ERK1/2 pathway. In recent years, activation of the ERK1/2 pathway has been shown to enhance the proliferation and migration of MSCs (Yan et al., 2017; Kim et al., 2024). In our study, inhibition of ERK1/2 phosphorylation by U0126 did affect the migration of BMSCs in the ELA group, suggesting that ELA promotes the migration of BMSCs in the H/R environment through ERK1/2 phosphorylation. The ERK1/2 pathway can regulate miRNA levels through multiple pathways, thereby regulating various cellular life activities (Sun et al., 2016). During osteogenic differentiation of MSCs, periostin (POSTN) could regulate the expression of miR-299-5p (Kaneda-Ikeda et al., 2020), while in another study, POSTN exerted cell migration proliferation and angiogenesis induced by ERK1/2 activation (Wang et al., 2016). We hypothesized that activation of ERK1/2 might regulate the expression of miR-299a-5p. In our study, ERK1/2 phosphorylation activated by ELA could regulate the expression of miR-299a-5p and its target gene Exo70, just as in tumor studies, Exo70 could be phosphorylated by ERK1/2 thereby promoting tumor metastasis (Mao et al., 2020). Our experiments culminated in the downregulation of miR-299a-5p through ERK1/2 phosphorylation and the upregulation of Exo70, which promoted the polymerization of F-actin and the migration of BMSCs to injured cardiomyocytes ultimately.

ERK1/2 is an essential downstream signaling factor of ELA/APJ, playing a pivotal role in cardiovascular development and protection from MI (Perjes et al., 2016). Our previous *in vitro* studies confirmed that ELA/APJ significantly attenuated apoptosis in MSCs through the ERK1/2 pathway (Fu et al., 2020). The majority of ELA functions were achieved through the APJ receptor (Dawid et al., 2021; Xi et al., 2023; Xu et al., 2023). Still, in embryonic stem cell studies, there was a possibility that the Receptor X played a specific function (Ho et al., 2015). To exclude the involvement of other receptors, we performed APJ receptors blockade, which resulted in a significant reduction in BMSCs migration. These findings demonstrate that ELA facilitates BMSCs migration through the APJ receptor in BMSCs. However, there are certain limitations in our study. Although we have identified the key mechanisms through which ELA facilitates the migration and homing of BMSCs to the injured myocardium, further studies are necessary to clarify the exact pathways that enable BMSCs to restore the impaired cardiac tissue subsequent to successful migration to the site of heart injury. Moreover, *in vivo* experiments are needed to assess the safety and efficacy of ELA pre-treatment in clinical applications.

## 5 Conclusion

In conclusion, our study reveals a novel mechanism through which ELA enhances BMSCs migration and homing to myocardial injury. ELA promotes the activation of ERK1/2 signaling, leading to the downregulation of miR-299a-5p and upregulation of Exo70, which in turn facilitates F-actin polymerization and cell migration. The study indicates that ELA exhibits potential as a therapeutic agent for enhancing stem cell therapy. Additionally, our results provide a robust theoretical foundation for the development and clinical implementation of ELA drugs, as well as the advancement of BMSCs transplantation in the treatment of AMI.

## Data Availability

The original contributions presented in the study are publicly available. This data can be found here: https://www.ncbi.nlm.nih.gov/sra/PRJNA1216908.

## Abbreviations

MSCs, Mesenchymal stem cells; BMSCs, Bone marrow mesenchymal stem cells; NRVMs, Neonatal rat ventricular myocytes; CVDs, Cardiovascular diseases; AMI, Acute myocardial infarction; ELA (Toddle), ELABELA; H/R, hypoxia-reoxygenation; MI/RI, Myocardial infarction/reperfusion injury; SD rats, Sprague-Dawley rats; PBS, Phosphate-buffered saline; BrdU, Bromodeoxyuridine; Exo70 (Exoc7), Exocyst complex component 7; Arp2/3, Actin-related proteins 2/3; ERK1/2, Extracellular regulated protein kinases 1/2; p-ERK1/2, Phosphorylated ERK1/2; t-ERK1/2, Total ERK1/2; APJ (APLNR), Angiotensin receptor-like 1; miRNAs, MicroRNAs; miR-299-5p (miR-299a-5p), microRNA-299-5p; siRNA, small interfering RNA; ECG, Electrocardiography; LVID; s, Left ventricular internal diameter in systole; LVID; d, Left ventricular internal diameter in diastole; LVEF, Left ventricular ejection fraction; LVFS, Left ventricular fractional shortening; LVESV, Left ventricular end-systolic volume; LVEDV, Left ventricular end-diastolic volume; RT-qPCR, Reverse transcription-quantitative polymerase chain reaction.
